# Construction of a Baculovirus-Silkworm Multigene Expression System and Its Application on Producing Virus-Like Particles

**DOI:** 10.1371/journal.pone.0032510

**Published:** 2012-03-05

**Authors:** Lunguang Yao, Shanshan Wang, Shuo Su, Ning Yao, Jian He, Li Peng, Jingchen Sun

**Affiliations:** 1 China-United Kingdom Nanyang Normal University-Rothamsted Research Joint Laboratory of Insect Biology, Henan Provincial Key Laboratory of Funiu Mountain Insect Biology, Nanyang Normal University, Nanyang, People's Republic of China; 2 Guangdong Provincial Key Lab of Agro-animal Genomics and Molecular Breeding, College of Animal Science, South China Agricultural University, Guangzhou, People's Republic of China; 3 School of Life Science and Engineering, Southwest Jiaotong University, Chengdu, People's Republic of China; 4 Guangzhou East Campus Lab Center, Sun Yat-sen University, Guangzhou, People's Republic of China; Commissariat a l'Energie Atomique(cea), France

## Abstract

A new baculovirus-silkworm multigene expression system named *Bombyx mori* MultiBac is developed and described here, by which multiple expression cassettes can be introduced into the *Bombyx mori* nuclear polyhedrosis virus (BmNPV) genome efficiently. The system consists of three donor vectors (pCTdual, pRADM and pUCDMIG) and an invasive diaminopimelate (DAP) auxotrophic recipient *E. coli* containing BmNPV-Bacmid (BmBacmid) with a homologous recombination region, an attTn7 site and a loxp site. Two genes carried by pCTdual are firstly inserted into BmBacmid by homologous recombination, while the other eight genes in pRADM and pUCDMIG are introduced into BmBacmid through Tn7 transposition and cre-loxp recombination. Then the invasive and DAP auxotrophic *E. coli* carrying recombinant BmBacmid is directly injected into silkworm for expressing heterologous genes in larvae or pupae. Three structural genes of rotavirus and three fluorescent genes have been simultaneously expressed in silkworm larvae using our new system, resulting in the formation of virus-like particles (VLPs) of rotavirus and the color change of larvae. The VLPs were purified from hemolymph by ultracentrifugation using CsCl gradients, with a yield of 12.7 µg per larva. For the great capacity of foreign genes and the low cost of feeding silkworm, this high efficient BmMultiBac expression system provides a suitable platform to produce VLPs or protein complexes.

## Introduction

Baculovirus expression system is one of the most ideal systems for routine production of recombinant eukaryotic proteins in insect cells, larvae and mammalian cells, which is widely-used in developing virus-like particles (VLPs) vaccine, displaying heterologous peptides or proteins, and transducing genes into mammalian cells [1], [2]. Besides the traditional *Autographa californica* multicapsid nucleopolyhedrovirus-*Spodoptera frugiperda* 9 (AcMNPV-Sf9) cell line system, another highly efficient baculovirus expression system, named *Bombyx mori* (silkworm) nucleopolyhedrovirus (BmNPV)-silkworm larvae/pupae system, has also been constructed to express heterologous genes. Compared with the AcMNPV-Sf9 system, the BmNPV-silkworm system provides enhanced expression level and pretty low cost in silkworm larvae or pupae, which shows promising industrialization future. Moreover, recent study has found that the N-acetyl glucosamine and galactose residues also exist in the N-glycan structures produced by silkworms, indicating silkworm larvae might be a useful host for producing human glycoproteins [3].

Until today, great efforts have been made for efficiently constructing recombinant BmNPV, including the BmNPV-based Bac-to-Bac system [4], [5], the mating-assisted genetically integrated cloning (MAGIC) method [6] and a method based on zero-background Tn7-mediated transposition in *E. coli* [7]. Other improvements relating to the baculovirus expression system also have been presented, such as utilizing cysteine protease and chitinase-deficient Bacmid to improve recombinant protein production and keep its stability [8], [9], as well as a transfectant-free method by directly infecting insect cells or injecting silkworm larva with invasive *E. coli* containing recombinant Bacmid [10], [11].

Normally, a foreign DNA fragment as large as 50 kb can be accommodated into the 130 kb dsDNA genome of baculovirus, which means several expression cassettes can be integrated into recombinant baculovirus. Thus, a multiple genes baculovirus expression system (MultiBac) has been rationally brought into the baculovirus vector for simultaneously expressing heterologous proteins [8]. The MultiBac system provides a powerful tool for over-expressing the low abundance protein complexes within cell for functional study. Another interesting multigene expression method in AcNMPV was performed using repeated homologous recombination and cre-loxp recombination to express up to 8 foreign proteins from 8 loci [12]. As some improvements on the construction of recombinant BmNPV have been made by us [6], [7], [10], [11], we are going to combine these different methods to establish another efficient MultiBac system based on BmNPV and silkworm. We have ever expressed rotavirus-like particles in cultured BmN cells by using the original vectors derived from MultiBac and the traditional method of recombinant baculovirus construction [13]. We question if this multigene system could work in silkworm larvae/pupae that shows promising future for large-scale production of protein complexes. In the present study, we successfully develop a novel silkworm-based baculovirus multigene expression system, in which multiple proteins can be expressed simultaneously to produce protein complexes or VLPs efficiently.

## Results

### Construction of BmNPV–silkworm multigene expression system

**Figure 1:**
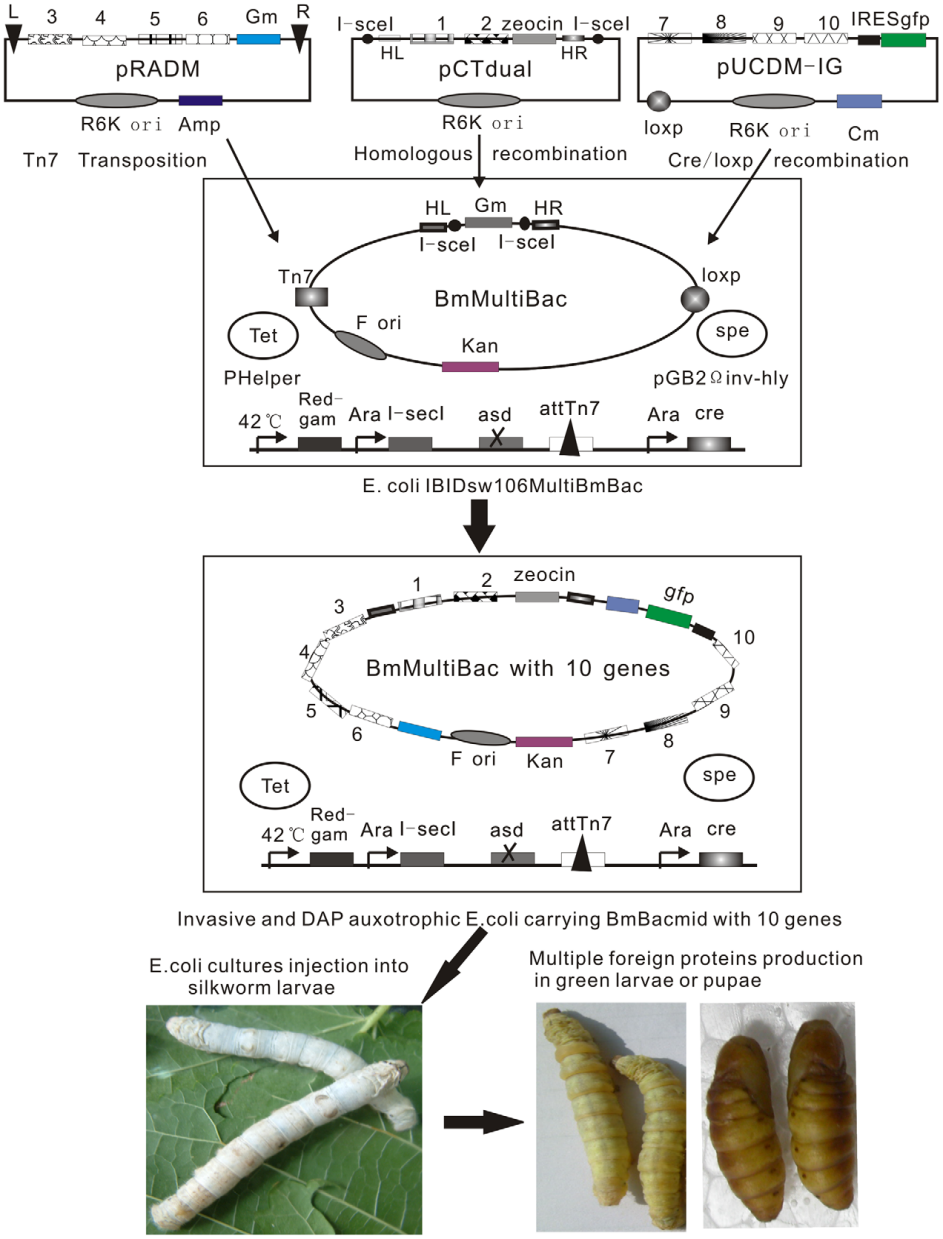
The flowchart of producing infective BmNPV expressing ten heterologous genes in silkworm larvae or pupae. The target genes are cloned into the three donor vector (pCTdual, pRADM and pUCDMIG) using usual method. The first two genes carried by pCTdual are inserted into BmBacmid through *I-Sce* I linearization and *red-gam* homologous recombination. Four genes in pRADM and the other four genes in pUCDMIG are then introduced into BmBacmid via Tn7 transposition and cre-loxp recombination, respectively. As a result, ten foreign expression cassettes and three antibiotic screening markers, as well as a GFP illumination marker are introduced into BmBacmid. The invasive and DAP auxotrophic *E. coli* carrying recombinant BmBacmid are injected into silkworm larvae at an appropriate dose. Consequently, recombinant BmNPV will be produced and multiple foreign genes will be expressed in green *B. mori* larvae or pupae.

Our BmNPV–silkworm multigene expression system consists of the modified recipient strain *E. coli* IBIDsw106MultiBmBac and three donor vectors including pCTdual, pRADM and pUCDMIG. All the donor plasmids contain the conditional replication R6kγ origin, the polh and p10 double promoters, and antibiotic resistance genes (Zeo^R^, Gm^R^ and Cm^R^) for screening, as well as some specific elements (homologous arms flanking *I-Sce* I sites, mini-Tn7 transposition arms and a P1-loxp site) for transferring expression cassettes into recipient MultiBmBacmid (Fig. 1).

*E. coli* IBIDsw106MultiBmBac is composed by the multiple functional host strain *E. coli* IBISW106Δasd and a modified BmBacmid named MultiBmBacmid. The invasive attTn7-blocked and DAP auxotrophic host strain contains the recombination genes *red* and *gam* under the control of a temperature sensitive repressor cI857, a tightly controlled arabinose-inducible *cre* gene and the *I-Sce* I expression cassette driven by *pBAD* promoter, as well as the Tn7 transposon helper plasmid pHelper (Tet^R^) and the invasive plasmid pGB2Ωinv–hly (Spe^R^). The modified BmBacmid has an attTn7 transposition recipient arms with the lacZ α fragment which replaces the original polyhedrin gene, a loxp site substituting the original *chitin* and *v-cath* genes, and a Gm^R^ cassette flanking two *I-sec* I sites which substitutes the original *p10* and *p74* genes (Fig. 1).

Resulting from the great capacity of foreign DNA fragment in BmNPV genome, up to ten heterologous genes can be easily incorporated into the BmBacmid and expressed at the same time with our multigene system. However, the first two genes in pCTdual must be integrated into MultiBmBacmid using homologous recombination to remove the Gm^R^, whereas the other eight genes can be introduced into BmBacmid through cre-loxp and mini-Tn7 transposon methods simultaneously. The efficiency of transferring genes from pCTdual to BmBacmid is 99.8%, and it is sufficient to ensure the positive recombinant identification with almost 100% success when combining the Gm sensitive testing [6]. The background of transposition using pRADM and attTn7-blocked host is negligible, and the white colonies are sure to be positive at 100% efficacy when the blue-white screening is still preserved [7]. However, the pUCDM-derivative integration into Bacmid is relatively less efficient (about 93%) through cre-loxp site-specific recombination method [8]. To fix this weakness, we introduced the IRES-egfp fragment into pUCDM to construct a new donor vector pUCDMIG, which contains the 5′-UTR IRES sequence from Rhopalosiphum padi virus [14] and a positive GFP marker. As the translation efficiency of IRES is about 3-fold weaker than that of the cap-dependent translation, the target gene upstream IRES-EGFP controlled by the same polh promoter will be expressed more efficient than the EGFP, showing the small IRES-EGFP cassette (1.3 kb) is a ideal illumination marker for recombinant baculovirus identification.

Moreover, a transfectant-free method is also included in the mutligene expression system, through which producing recombinant BmNPV in silkworm becomes simple and rapid by intrahemocoelic injection with invasive diaminopimelate auxotrophic *E. coli* containing MultiBmBacmid [11].

### Production of infective recombinant BmNPV expressing multiple foreign genes in *B. mori* larvae

**Figure 2:**
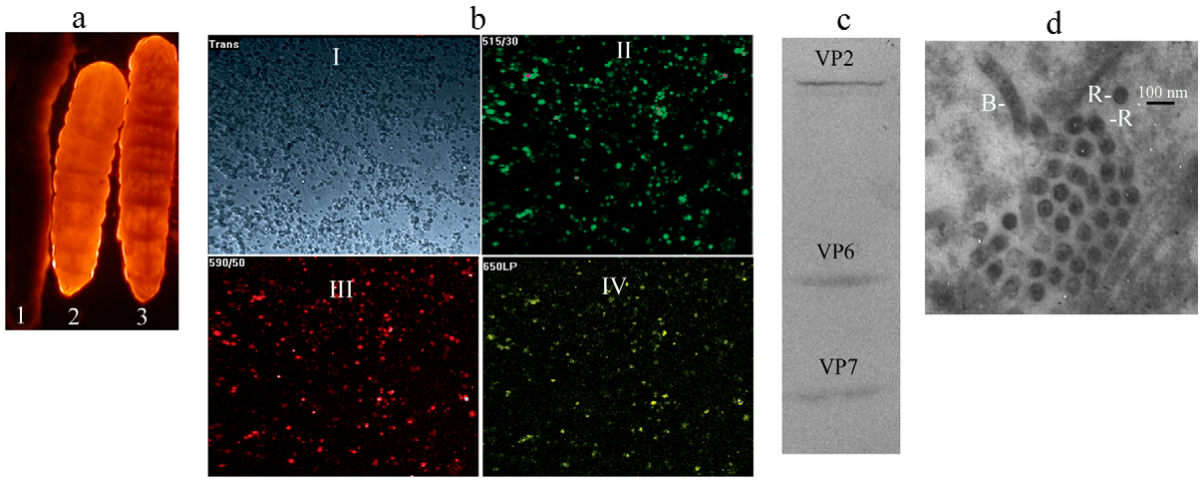
Multiple genes expression and rotavirus-VLPs production in silkworm. (a) The larvae of six days post injection were observed using the fluorescence detection device. 1: the mock injected larvae; 2&3: the larvae injected with invasive and DAP auxotrophic *E. coli* carrying BmBacmid with six genes including *egfp*, *dsRed*, *eyfp*, *vp2*, *vp6* and *vp7* at a dose of 8.0×10^8^ cells per larva. (b) The hemolymph from a red larva was observed using laser confocal microscope. The images of hemocytes were taken at the bright (trans) channel (I), GFP (515 nm) detection channel (II), DsRed (590 nm) channel (III) and YFP (530 nm) channel (IV). (c) Western blot analysis of the hemolymph from the red larvae using anti-VP2, anti-VP6 and anti-VP7 rabbit antiserums. (d) The EM image of hemocytes collected from red larvae. R: the round Rotavirus-VLPs; B: the rod shape baculovirus particle, bar = 100 nm.

The invasive DAP auxotrophic *E. coli*, which contains recombinant MultiBmBacmids carrying six foreign genes including *egfp*, *dsRed*, *eyfp*, *vp2*, *vp6* and *vp7*, was injected into silkworm larval hemocoel with 15 µl overnight cultures at a 10 fold dilution (OD_600_≈2.0, total cell number≈10^8^) per larvae. Most of the injected larvae (90%) turned red in sunlight six days post injection, which also displayed red when observed with the gel imaging system (Fig. 2a). The hemocytes were found to be expressing GFP, DsRed and YFP simultaneously when observed under a laser confocal microscope (Fig. 2b). It indicated that infective recombinant BmNPV has been generated and the three fluorescence proteins were also expressed successfully in *B. mori* larvae. The injected silkworm larvae displaying red as their major color due to the tetramers formed by DsRed fluorescence proteins expressed in the *B. mori* larvae.

### Production of rotavirus-VLPs in silkworm larvae

In addition to the three fluorescence proteins, the three structural proteins (VP2, VP6 and VP7) of rotavirus were also successfully expressed in silkworm larvae according to the western blotting experiment (Fig. 2c). Furthermore, the round virus like particles were found in the EM specimen, which meaned VP2, VP6 and VP7 were coexpressed in silkworm larvae and had self-assembled into VLPs (Fig. 2d). The results above indicated multiple genes were able to be co-expressed in silkworm simultaneously using our new BmMutiBac expression system.

**Figure 3:**
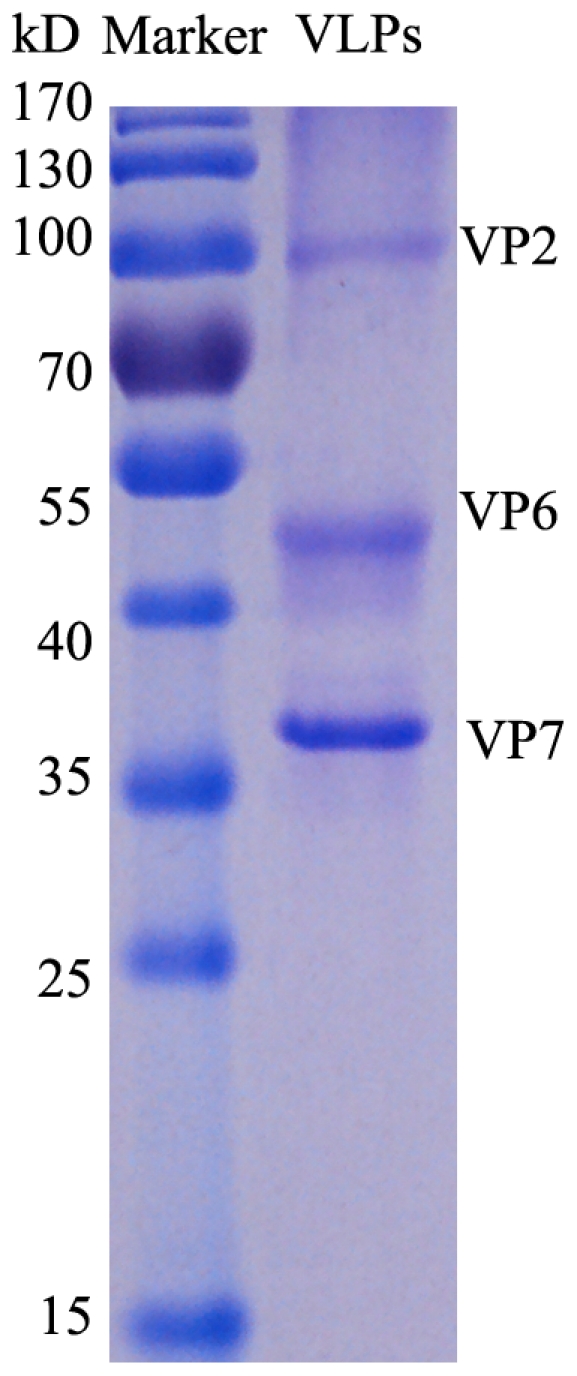
SDS-PAGE analysis of purified VLPs of rotavirus from silkworm. 5 µg VLPs was loaded on 12% SDS-PAGE gel. Lane Marker: standard protein marker. lane VLPs: purified VLPs from silkworm hemolymph. The three bands VP2, VP6 and VP7 were labelled.

About 150 ml hemolymph was collected from 500 red larvae. The rotavirus VLPs were purified from the larval hemolymph by traditional ultracentrifugation using CsCl gradients. Two major bands were recovered for VLPs detection by TEM. One band was composed of baculoviral particles in rod shape, the other was made of round rotavirus VLPs. The result of SDS-PAGE proved the VLPs were constructed by three viral coat proteins including VP2, VP6 and VP7 as expected (Fig. 3). The total protein content in the purified VLPs from the hemolymph collected from 500 larvae was 6.35 mg, which meaned the yield of VLPs was 12.7 µg per larval hemolymph. Obviously this BmNPV-silkworm multigene expression system provides an economic and efficient solution for producing antiviral vaccine derived from VLPs.

## Discussion

Baculovirus expression system is always a popular tool for expressing recombinant proteins for its high expression level and the great convenience of cell culture. Recombinant baculoviral genome is able to receive 50 kb foreign DNA fragment, which enables the possibility to express different genes or multiple copies of one gene simultaneously. Berger and his colleagues have established a multigene expression system based on AcMNPV-Sf9 cell line and successfully expressed a transcript factor TFII complex [8]. Comparing to AcMNPV-Sf9 system, BmNPV-silkworm larvae expression system is more attractive because of the lower cost of breeding silkworm and higher expression level. Following this idea, we successfully constructed a BmNPV-silkworm multigene expression system, in which six foreign genes are introduced into BmBacmid through homologous recombination, zero background Tn7 transposition and cre-loxp site-specific recombination. In the model shown in Fig. 1, four expression cassttes can be inserted into the donor vector pRADM or pUCDMIG using isocaudamer technique described Berger and his colleagues, as a result, up to ten genes can be introduced into the recombinant baculovirus.

Upon the power of its three donor vectors (pCTdual, pRADM and pUCDMIG), the efficiency of transferring multiple foreign genes into BmBacmid keeps at an extremely high level. A positive GFP marker is also introduced into the BmBacmid to monitor the protein expression using the donor vector pUCDMIG, which contains IRES-EGFP sequence [14]. The small IRES-GFP fragment has little influence on the capacity of vector and the expression level of the upstream gene, proving it is a good illumination marker [15].

Furthermore, a transfectant-free method is also presented in our multigene expression system, which immensely reduces the procedures and working time by intrahaemocoelic injection with invasive diaminopimelate auxotrophic *E. coli* containing MultiBmBacmid rather than usual transfection method.

We have used this new system to co-express three structural genes of rotavirus in silkworm for VLPs production, and the yield of purified VLPs from hemolymph was up to 12.7 µg per larva. The productivity will be increased if we try to co-express two copies of the viral structional genes or improve the VLPs purification method. As VLPs are not only assembled in hemolymph, but also in other larval tissues, it is necessary to develop an efficient method for purification VLPs from the whole lavae in the next study. Recently, Noad et al used a kind of high efficient repeated homologous recombination method to express eight genes at eight different loci in Bacmid, and all the target genes at different loci were expressed at high level [12]. In our system, two or four expression cassettes need to incorporate into one locus for expressing multiple genes simultaneously. According to our previous experience, there was no significant difference when expressing two genes in one locus or in two separated loci. However, It is necessary to study whether expression level is hampered by the incorporation of more than two genes in one locus in our future work.

In brief, up to ten heterologous genes or ten copies of one gene can be co-expressed in silkworm efficiently with our multigene expression system, which provides an economic and rapid platform for both recombinant multiprotein production and multigene transfer applications. We are going to express macromolecular complexes in silkworm to study their molecular structure and function using the system in the future.

## Materials and Methods

### Bacterial strains, plasmids, viral Bacmid, reagents and larvae

*E. coli* DH10B, BW23474 and TOP10 were used for the propagation of BmBacmid, R6kγ origin derived plasmids and other pUC derived plasmids, respectively. *E. coli* SW106 was provided by Prof. Copeland [16]. Plasmids pML291, pcp15 and pcp20 were gifts from Dr. Li [17], and spectinomycin (*spe*)-resistance plasmid pGB2Ωinv–hly (containing both *hly* and *inv* genes) was provided by Prof. Courvalin [18]. Plasmids pBlock, pRCDM and pCTdual, as well as the modified BmBacmid with gentamycin resistance gene between two *I-sce* I sites were constructed at our previous study [6], [7]. Plasmids pUCDM and pFBDM were from Prof. Richmond [8]. Plasmid pBac-IR-GFP containing the 5′-UTR internal ribosome entry site (IRES) sequence of Rhopalosiphum padi virus was provided by Prof. Wu [14].

Pfu*Taq*, restriction enzymes and T4 DNA ligase were purchased from NEB (New England Biotechnologh, England), while DL-α-ε Diaminopimelic acid (DAP) was bought from Sigma (cat. D1377, USA). Low salt (LS) medium (10 g of tryptone, 5 g of NaCl and 5 g of yeast extract in 1 liter of broth, pH7.5) was used for cloning and growing the plasmids containing zeocin resistance gene. Silkworm variety named Chinese Ming-zhu from Yunyang Silkworm Breeding Farm (Nanyang City, China) was fed with mulberry leaves.

### Generation of multifunctional strain *E. coli* IBISW106Δasd

The original *E. coli* strain SW106 (mcrA Δ(mrr-hsdRMS-mcrBC) ΔlacX74 deoR endA1 araD139 Δ(ara, leu) 7697 rpsL recA1 nupG ϕ80dlacZΔM15 [λc1857 (cro-bioA)<>Tet] (cro-bioA)<>araC-PBAD Cre ΔgalK) carries a tightly controlled arabinose-inducible *cre* gene and the recombination genes *red* and *gam* under the control of a temperature sensitive repressor cI857. The *asd* gene of *E. coli* encodes aspartic semialdehyde dehydrogenase, an enzyme involved in lysine, threonine and methionine biosynthesis [19]. Deletion of *asd* gene from *E. coli* genome will result in DAP auxotrophy and defective cell wall synthesis. The *asd* gene was deleted from SW106 genome by homologous recombination as described previously [10].

A 1.6 kb kanamycin cassette flanking FRT sequences and 50 bp *asd* gene homologous recombination arms was amplified from pcp15 with the primers asd50F: GAG ACC GGC ACATTT ATA CAG CAC ACA TCT TTG CAG GAA AAA AACGCT TA GAATTC GAGCTC GGTACC C GGG and asd50R: CTCCTG TAT TAC GCA CTA ACA GGG GCG GCA TCG CGCCCC AGA TTT AAT GA CTT A AGCTTAAAA GCGCTCTGAA (50 bp homologous sequences underlined). The PCR product was firstly digested with *Dpn* I to remove the possible plasmid template, and then was electroporated into the 42°C induced electro-competent *E. coli* SW106 prepared according to previous work [16]. The transformed cells were spread on agar plate supplemented with 50 µg/ml kanamycin and 0.5 mM DAP and cultured at 32°C overnight. The positive colonies grown on the kan/DAP plate were picked and identified by PCR. Then the kanamycin cassette was removed from the *E. coli* genome by Flp-mediated excision *in vivo* using plasmid pcp20 [20]. The target DAP auxotrophic strain was named as *E. coli* SW106Δasd.

The *I-Sce* I expression cassette driven by pBAD promoter was introduced into the *E. coli* SW106Δasd genome through Tn7 transposition. Briefly, the *Eco*R I-*Kpn* I (filled in) fragment from pML291 containing ParaBAD-I-SceI-FRT-npt-FRT was cloned into the *Sac* II and *Bam*H I sites (filled in) of pBlock to replace the FRT–zeocin-FRT fragment. The target recombinant plasmid was transformed into *E. coli* SW106Δasd containing transposition helper plasmid pHelper to block the attTn7 site in *E. coli* genome as described previously [7], [10]. The kanamycin cassette (npt) was removed by Flp-mediated excision *in vivo* using plasmid pcp20. As a result, the *I-Sce* I expression cassette was introduced, while the attTn7 site of SW106 genome was blocked simultaneously. Plasmid pGB2Ωinv–hly which contains *inv* and *hly* expression cassettes was transformed into the modified *E. coli* strain to generate the invasive, attTn7 blocked, DAP auxotrophic, *I-Sce* I homing endonuclease and *cre* recombinase expressing *E. coli* IBISW106Δasd.

### Construction of the recipient MultiBmBacmid

BmBacmid-Gm, constructed previously by introducing a Gm^R^ cassette flanking two *I-Sce* I sites into the *p10* and *p74* locus of the original BmNPV-Bacmid through homologous recombination, was transformed into *E. coli* SW106 [6]. After the induction of lambda *red-gam* recombinase at 42°C for 15 minutes, the electro-competent cells of SW106 containing BmBacmid-Gm were prepared and stored as previous description [16]. A 2.6 kb fragment containing both *chitA* (accession number NC001962; GeneID: 1724489) and *v-cath* (accession number NC001962;GeneID:1724490) was amplified from BmBacmid using the primers chitAF (AGTATACGCGTTGAGCAAGTCGCCGTTATCGG) and vcathR (AGTATACCCTGAAAAATCCGTCCTCTCCCC, *Bst*Z17I underlined). The PCR product was cloned into pMD18-simpleT vector (TaKaRa, Japan) to get pST-chita-vcath, then the recombinant plasmid was digested with *Bst*B I and *Sac* II to remove the 1.9 kb fragment which contains 1.4 kb coding sequence of *chitA* and 0.5 kb coding sequence of *v-cath*. The blasticidin resistance cassette (*Bsd*, accession number BAF91001 ) flanking loxp sequence was amplified from pIBV5His (Invitrogen) using the primers BsdloxpF ( TTCGA*A* *TAACTTCGTATAGCATACATTATACGAAGTTAT*GCAGCACGTGTTGACAATT, *Bst*B I underlined, loxp site italics) and BsdloxpR ( CCGCGG *ATAACTTCGTATAATGTATGCTATACGAAGTTAT*GTCAGTCCTGCTCCTCGG, *Sac* II underlined). The PCR product was first cloned into pMD18-simple T vector for sequencing, and then the plasmid was digested with *Sac* II and *Bst*B I. The released loxp-Blastidin-loxp fragment was ligated into the *Sac* II/*Bst*B I-digested pST-chita-vcath described above. The resultant plasmid was digested with *Bst*Z17 I and the released target fragment containing the *Bsd* cassette flanking loxp sequence and homologous arms was transformed into the 42°C -induced electro-competent cells of *E. coli* SW106/BmBacmid-Gm prepared according to previous report [13].

The positive candidates grown on kanamycin/blasticidin LS agar plate were further confirmed by colony PCR using the primers chitAF and vcathR. Following that, the blasticidin cassette was removed from BmBacmid-Gm by cre-loxp recombination induced by L-arabinose, therefore only one loxp site was left in the BmBacmid-Gm. The resultant BmBacmid-Gm-loxp was named MultiBmBacmid, which contains an attTn7 transposition site, a loxp site and a homologous recombination site. The MultiBmBacmid was transformed into the multifunctional *E. coli* IBISW106Δasd to generate target strain *E. coli* IBIDsw106MultiBmBac.

### Construction of donor vectors

The ampicillin resistance gene (Amp^R^) was amplified from pFBDM (accession number DJ417502) using the primers AmpF (AAATTCGAA TTTCAGGTGGCACTTTTCGG, *Bst*B I underline) and AmpR (AAATTCGAA TTTCTACGGGGTCTGACGCT). The 1.1 kb PCR product was first digested with *Bst*B I, and then cloned into the same site of pBlock to replace the chloramphenicol resistance gene (Cm^R^) for constructing pBlockA. The gentamycin resistance gene (Gm^R^) and the two promoters (polh and p10) were obtained from *Sac* II/*Avr* II-digested pFBDM. The target *Sac* II/*Avr* II fragment was cloned into the same sites of pBlockA to replace the zeoFRT fragment, forming the Tn7 transposon donor vector pRADM (Fig. 1; accession number : JN596961). IRES fragment (accession number AX376819)was amplified from pBac-IR-GFP using the primers iresF (AAATTCGAAGATAAAAGAACCTATAATCC, *Bst*B I underlined) and iresR (AAATCTAGATATAAATAGATAAAGCTAA, *Xba* I underlined). The 580 bp PCR product was digested with *Xba* I and *Bst*B I, and then cloned into the same sites of pUCDM (accession number DJ417503 ) to produce pUCDM-IRES. *Egfp* cDNA was amplified from pEGFP-1 (Clontech; accession number U55761) using the primers egfpspeI (AAACTAGTAACATGGTGAGCAAGGGCGAG, *Spe* I underlined) and egfppstI (AAACTGCAGTTACTTGTACAGCTCGTC, *Pst* I underlined). The 0.7 kb PCR product was finally cloned into pUCDM-IRES via *Xba* I and *Pst* I to create pUCDMIG (accession number : JN596960) containing the IRES-EGFP fragment (Fig. 1).

*DsRed* (accession number ACJ05619) from pDsRed2-1 was cloned into pCTdual (accession number : JN596959) via *Sma* I and *Xho* I to generate pCTdual-Red, in which *DsRed* was driven by p10 promoter. The *vp2* gene of human rotavirus (accession number AB022766 ) was amplified from the viral RNA by RT-PCR using the primers vp2F (AAAGGATCCACCATGGCGTACAGGAAGCGCGGA, *Bam*H I underlined) and vp2R (AAAGTCGACTCCACAGTGGGGTTGGCGTTTACA, *Sal* I underlined). The 2.7 kb PCR product was then cloned into pCTdual-Red through *Bam*H I and *Sal* I to form pCTdual-Red-vp2, in which the *vp2* gene was driven by polh promoter while *DsRed* was controlled by p10 promoter. *Eyfp* (accession number AAV97917)from pEYFP-1 was cloned into the *Sma* I and *Xho* I sites of pRADM to get pRADM-YFP. The v*p6* gene of human rotavirus (accession number AB022768 )was amplified from the viral RNA using the primers vp6F (AAAGGATCCACCATGGAGGTTCTGTACTC) and vp6R (AAAGTCGACTCACTTAATCAACATGCTTC). The 1.2 kb PCR product was cloned into pRADM-YFP via *Bam*H I and *Sal* I to generate pUCDM-YFP-vp6, in which the *vp6* and *eyfp* were driven by polh and p10 promoter, respectively. The 1.0 kb *vp7* gene of human rotavirus (accession number AB018697) was amplified using the primers VP7F (AAAGGATCCACCATGGGCTATGGTATTGAATATAC) and VP7R (AAAGTCGACCTATACTCTGTAGTAAAA), and the RT-PCR product was cloned into pUCDMIG via *Bam*H I and *Sal* I to form pUCDMIG-vp7, in which the *vp7* and *egfp* were controlled by the same polh promoter but the translation of *egfp* was medicated by IRES element.

### Introduction of multiple genes into BmBacmid

Both *vp2* and *DsRed* genes were introduced into MultiBmBacmid through homologous recombination according to previous study [5]. In practice, the plasmid pCTdual-Red-vp2 was transformed into the 42°C and L-arabinose induced electro-competent cells of *E. coli* IBIDsw106MultiBmBac. The colonies grown on Zeocin/Kan/Tet/spe/DAP were picked for further Gm susceptive detection. The positive zeocin resistant and Gm susceptive strain was named *E. coli* SW106BmMutiBac-Red-VP2. The *vp6* and *eyfp* were introduced from pUCDM-YFP-vp6 into BmBacmid (BmMutiBac-Red-VP2) by Tn7 transposition as described previously [6]. Furthermore, the *vp7* and IERS-egfp were introduced from pUCDMIG-vp7 into BmBacmid (BmMutiBac-Red-VP2-YFP-VP6) through cre-loxp site specific recombination according to previous report [8].

Finally, the target DAP auxotrophic *E. coli* containing the recombinant BmBacmid carrying six foreign genes (BmBacmid-gry267) and the invasive plasmid pGB2Ωinv–hly was injected into 5^th^ instar *B. mori* larvae at a dose of 10^8^ cells per larva according to our previous procedure [11] . The injected larvae were monitored until they turned green, red or yellow.

### Production of VP2, VP6 and VP7 antiserum

For preparation of antiserum, truncated VP2, VP6 and VP7 proteins were expressed and purified from *E. coli*. Partial sequences of *vp2* (from start codon ATG +1–465), *vp6* (+1–510) and *vp7* (+1–496) were respectively amplified from the plasmids pCTdual-Red-vp2, pUCDM-YFP-vp6 and pUCDMIG-vp7 using the following primers: SVP2F (CCGGAATTCATGGCGTACAGGAAGCGCG, *Eco*R I underlined), SVP2R (CCGCTCGAGATTTGCTCGGTAGATTGG, *Xho* I underlined), SVP6F (CCGGAATTCATGGAGGTTCTGTACT), SVP6R (CGCTCGAGTTGTGATCTGTTCAGTGT), SVP7F (CCGGAATTCATGGGCTATGGTATTG) and SVP7R (CCGCTCGAGGCACAGCCATTCGTTCAG). The PCR products were respectively cloned into the *Eco*R I and *Xho* I sites of pET32a (Novagen) in frame with the His tag at N-terminus to form three recombinant pET32a expression vectors, which were transformed into *E. coli* BL21 (DE3) competent cells later.

The three truncated VP2,VP6 and VP7 proteins were expressed, isolated and affinity-purified following the instructions in the *His-tag* fusion protein purification manual (FPLC of GE Health). New Zealand white rabbits were inoculated with the purified proteins to produce polyclonal antibodies using the standard procedures [21].

### Fluorescence microscopy

The silkworm larvae injected with *E. coli* containing recombinant BmBacmid–gry267 were observed using Landun 652 visible light gel imaging system. In this system, the blue filter is used to generate blue light (about 490 nm wavelength) for excitation, and the orange filter is used to observe green, yellow and red fluorescence simultaneously. The hemolymph was collected from the larvae injected with *E. coli*, and observed under a Nikon laser scanning confocal microscope. The excitation wavelengths used to excite EGFP, EYFP and DsRed were 488 nm, 514 nm and 543 nm, respectively. Correspondingly, the 515 nm, 530 nm and 590 nm channel were used to detect the green, yellow and red fluorescent proteins, respectively.

### Western blot analysis and electron microscopy

The hemolymph of the red silkworm larva was collected, into which 5 mM phenyl-thiourea was added to prevent melanization. The hemocytes were harvested by centrifugation, and then lysed by incubation with lysis buffer (1% Triton-X 100 in 10 mM Tris-Cl, pH 7.4) on ice for 30 minutes. The cell lysates were separated in 10% SDS-PAGE, followed by western blot analysis with anti-VP2, anti-VP6 and anti-VP7 rabbit antiserums (diluted 2000×), as well as appropriate secondary antibody (alkaline phosphatase-conjugated goat anti-rabbit IgG, GE Health) according to the manufacturer's protocol.

The harvested hemocytes pellets were fixed in 4°C PBS buffer (pH 7.2) containing 2.5% glutaraldehyde for 2 h. After rinsing with PBS buffer, the samples were fixed in 1% osmium, and dehydrated through a series of graded ethanol baths. Then the dehydrated samples were transferred to propylene oxide for 20 min, and embedded in Epon812 for ultrathin sectioning. Ultrathin sections were stained with 2% uranyl acetate and 1% lead citrate, and then they were examined under a JEM-100SX transmission electron microscope (TEM). For purified VLPs observation, a drop (3 µl) of VLPs sample recovered from the band was applied onto carbon coated 400 mesh copper grid and negatively stained with 2% uranyl acetate. Finally, the dried grins were examined using the transmission electron microscope JEM-100SX.

### VLPs purification and quantification

The hemolymph of 500 red larvae infected with recombiant virus BmBacmid-gry267 was collected, into which 5 mM phenyl-thiourea was added to prevent melanization and 50 µl of Protease Inhibitor Cocktail (Sigma P8849) was added to inhibit the proteinases. The collected hemolymph was then treated by sonication for 3 min (periods of 10 s, separated by pauses 5 s). The cell debris were removed by centrifugation at 8,000 rpm (Beckman, Rotor JA-25.50) for 10 min at 4°C. The supernatant was then ultra-centrifugated upon a 20% sucrose cushion at 2,4000 rpm (Beckman, Rotor JA-25.50) for 1 h at 4°C.The pellet was resuspended in 10 ml D-PBS. Following, 4 g of CsCl was added for the preparation of a CsCl gradient. The suspension was then ultra-centrifuged at 3,5000 rpm (Beckman, Rotor SW41)for 18 h at 4°C. The bands were recovered separately and a drop of which was examined by electro microscope as described above, as well as western blot was performed for analysis of the VLPs construction. The total protein content in purified VLPs was determined by using BCA protein kit from Pierce, followed by running a SDS-PAGE gel.
